# Metrology of morphological response of Siberian elm to drought stress: increased stomatal pore depth

**DOI:** 10.1186/1753-6561-5-S7-P90

**Published:** 2011-09-13

**Authors:** Ki Woo Kim, Go Eun Park, Don Koo Lee

**Affiliations:** 1 School of Ecological and Environmental System, Kyungpook National University, Sangju, 742-711, Korea; 2 Department of Forest Environmental Science, Seoul National University, Seoul, 151-742, Korea

## Background

Structural characteristics of stomata consist of stomatal shape, density, depth, and pore dimension. Commonly called Siberian elm, the species *Ulmus pumila* L. is a fast-growing and small to medium-sized tree. Due to the superb adaptations to the harsh conditions of the Gobi Desert, the trees have been preferentially planted in Mongolia. It is worthwhile to investigate the morphological characteristics of the tree species that are tolerant to drought stresses in such arid areas.

## Materials and methods

Based on average annual precipitations, two types of leaf specimens were collected from Korea, China, and Mongolia: (i) leaves under normal environmental conditions and (ii) leaves under arid conditions. Leaf stomatal characteristics of Siberian elm were investigated by electron microscopy and white light scanning interferometry [1].

## Results and conclusions

**Figure 1:**
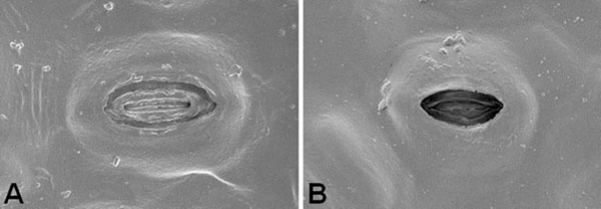
Field emission scanning electron micrograhs of stomata of Siberian elm. (A) Stoma under normal conditions. (B) Stoma under arid conditions.

**Figure 2:**
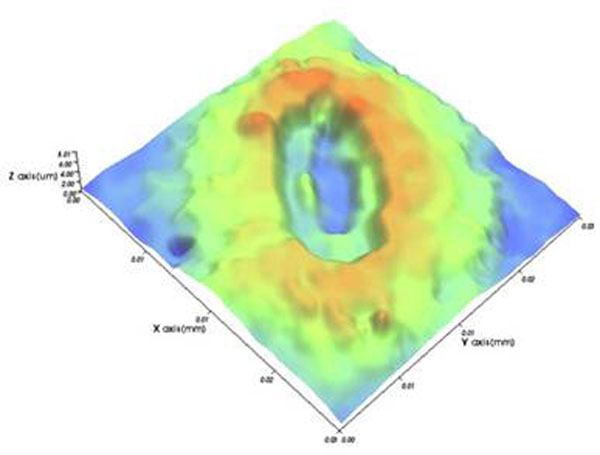
Three-dimensional surface plot of a stoma under arid conditions by white light scanning interferometry.

Field emission scanning electron microscopy revealed stomata on the lower leaf surface of the tree species. Measured as ca. 30 micrometers in width, the stomata appeared to be oval in shape. In-lens secondary electron imaging by a coaxial annular type detector showed a difference in depth from epidermis to pore between the two types of leaves. Leaf stomata under arid conditions appeared to have higher levels of depth from epidermis to pore than ones under normal conditions (Figure 1). Line profile analysis by white light scanning interferometry allowed for the nondestructive measurement of the stomatal dimension (Figure 2). The depth from epidermis to pore of stomatal complexes under normal conditions was ca. 1.79±0.13 micrometers, whereas that under arid conditions was ca. 2.12±0.08 micrometers. In addition, higher levels of surface roughness were observed in stomata under arid conditions than those under normal conditions. These results suggest that increased stomatal pore depth would be responsible for the adaptations of the tree species to arid conditions. Furthermore, such architectural differences in stomatal dimension could be quantitatively analyzed by complementary microscopy.
